# Modelling sociodemographic factors that affect malaria prevalence in Sussundenga, Mozambique: a cross-sectional study.

**DOI:** 10.12688/f1000research.75199.1

**Published:** 2022-02-14

**Authors:** Joao Ferrao, Dominique Earland, Anisio Novela, Roberto Mendes, Marcos Ballat, Alberto Tungadza, Kelly Searle

**Affiliations:** 1 Engineering & Agriculture, 1Instituto Superior de Ciências e Educação a Distância, Beira, Sofala, Mozambique; 2 School of Public Health, University of Minnesota, Twin City, Minnesota, USA; 3 Hospital Distrital de Sussundenga, Direccao Distrital de Saude, Susssundenga, Manica, Mozambique; 4 GIS - Faculdade de Economia e Gestao, Universidade Catolica de Mocambique, Beira, Sofala, Mozambique; 5 Faculdade de Engenharia, Universidade Catolica de Mocambique, Chimoio, Manica, Mozambique; 6 Faculdade de Ciências de Saúde, Universidade Católica de Moçambique, Chimoio, Manica, Mozambique; 7 School of Public Health, University of Minnesota, Twin City, Minessota, USA

**Keywords:** sociodemographic, social determinants of health, malaria, prevalence, Sussundenga

## Abstract

**Background**: Malaria is still one of the leading causes of mortality and morbidity in Mozambique with little progress in malaria control over the past 20 years. Sussundenga is one of most affected areas. Malaria transmission has a strong association with environmental and sociodemographic factors. The knowledge of sociodemographic factors that affects malaria, may be used to improve the strategic planning for its control. Currently such studies have not been performed in Sussundenga. Thus, the objective of this study is to model the relationship between malaria and sociodemographic factors in Sussundenga, Mozambique.

**Methods:** Houses in the study area were digitalized and enumerated using Google Earth Pro version 7.3. In this study 100 houses were randomly selected to conduct a community survey of *Plasmodium* *falciparum* parasite prevalence using rapid diagnostic test (RDT). During the survey, a questionnaire was conducted to assess the sociodemographic factors of the participants. Descriptive statistics were analyzed and backward stepwise logistic regression was performed establishing a relationship between positive cases and the factors. The analysis was carried out using SPSS version 20 package.

**Results:** The overall *P. falciparum* prevalence was 31.6%. Half of the malaria positive cases occurred in age group 5 to 14 years. Previous malaria treatment, population density and age group were significant predictors for the model. The model explained 13.5% of the variance in malaria positive cases and sensitivity of the final model was 73.3%.

**Conclusion:** In this area the highest burden of *P. falciparum* infection was among those aged 5–14 years old. Malaria infection was related to sociodemographic factors. Targeting malaria control at community level can combat the disease more effectively than waiting for cases at health centers. These finding can be used to guide more effective interventions in this region.

## Background

Malaria is a serious and sometimes fatal disease caused by a *Plasmodium* spp. parasite that commonly infects *Anopheles* spp. mosquitos which feed on humans. Although malaria can be a deadly disease, infection and death can be prevented. ^1^ Almost half of the world’s population lives in areas at risk of malaria transmission. Six countries account for more than half of all malaria cases worldwide and Mozambique is among them. ^2^

In Mozambique, a country in Sub-Saharan Africa, with a population of over 30 million, malaria is one of the leading causes of mortality and morbidity. In 2018, Mozambique recorded the third largest number of malaria cases in the world, accounting for 5% of all cases. ^3^

The country has made little progress in malaria control. Indoor residual spraying (IRS), insecticide treated bed nets (ITNs), and parasitological diagnosis in health facilities using rapid diagnostic test (RDTs) with effective artemisinin combination therapy (ACT) are the forms of malaria intervention currently being used. The entire country uses RDTs with ACT as the standard of care in public health facilities and ITNs are only available at antenatal clinics, indicated for pregnant women and children under five. ^4^

Manica Province in central Mozambique has the second highest number of malaria incidences in the country. In the first quarter of 2020, there were 1,039,283 recorded cases with an incidence of 371 per 1000 inhabitants. ^5^ Sussundenga village, in Manica Province is one of most affected areas, with 31,397 malaria cases reported in 2019.

Malaria risk, disease severity, and clinical outcome depend on environmental, sociodemographic, economic, and behavioral factors. ^6^^–^^9^ A study in Chimoio, the provincial capital of Manica, close to Sussundenga Village, modelled the influence of climate on malaria occurrence. The study indicated that selected environmental characteristics accounted for 72.5% of malaria incidences, implying that non-environmental factors such as sociodemographic, economic, cultural and behavioral traits would account for the rest. ^10^

While Mozambique is a country with one of the highest incidences and prevalence of malaria in the region and, it accounts for nearly half of childhood deaths, little is known about the epidemiology to inform appropriate and effective interventions. This is one of two major barriers to expanding control measures in the country with the other being limited funding.

In the country, malaria transmission occurs all year round and, the knowledge of sociodemographic factors that affect malaria is crucial for informing the implementation of the most appropriate and effective malaria interventions to achieve control. In Sussundenga no studies are known in this field. Therefore, the objective of this study was to model the relationship between malaria and sociodemographic factors in Sussundenga’s rural municipality.

## Methods

### Study area

**Figure 1.**
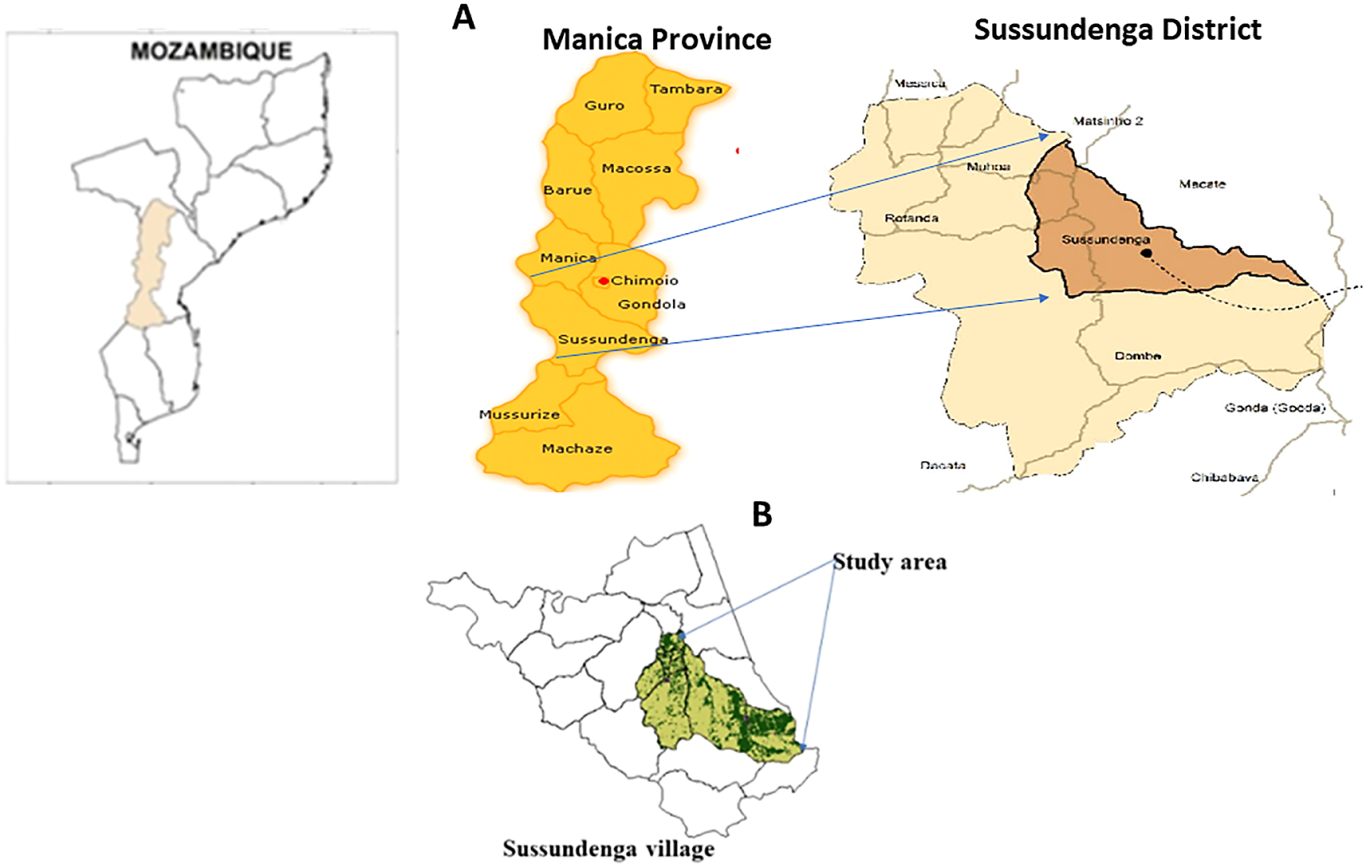
Study area. A. Map of Mozambique, Manica province and Sussundenga district: adapted from National Cartography and Remote Sensing Centre ( CENACARTA). ^11^ B. Sampled site in Sussundenga village: adapted from CENACARTA.

The village of Sussundenga is a rural, agrarian community 40 km from the Zimbabwe border, and is 40 km from the provincial capital of Chimoio ( Figure 1).

Sussundenga has an estimated population of 31,429 inhabitants, 47% males and 53% females. The age distribution is: 19.5% from 0 to 4 years old, 29.9% from 5 to 14-years-old, 20.5% from 15 to 24 years old and 30.1% with over 24 years old. The village is divided administratively in 17 residential areas (neighborhoods). ^11^

The climate is tropical with an average annual precipitation of 1,200 mm. The rainy season occurs from November to April. The average minimum temperature is 6.3°C in the month of July and the average maximum temperature is 38.9°C in the month of January and the annual average is 21.2°C. ^12^

### Data collection

**Figure 2.**
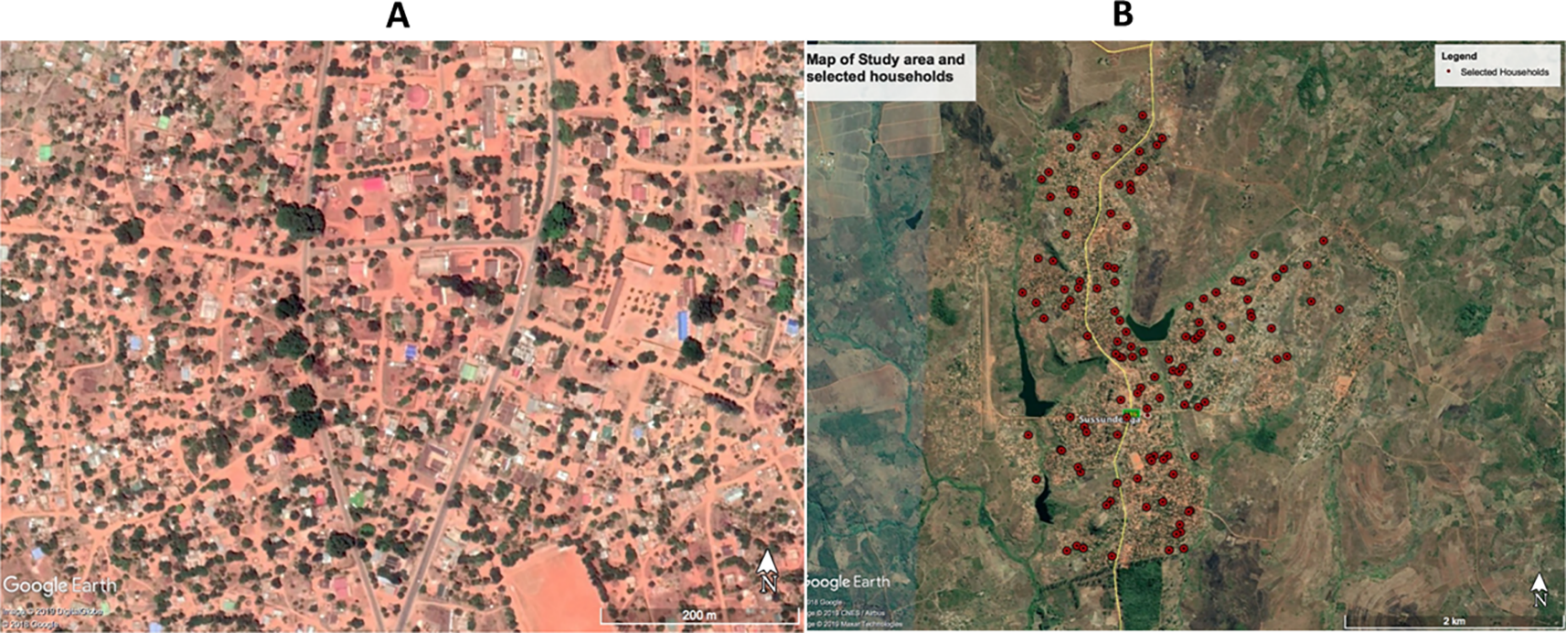
A. High-resolution imagery of Sussundenga village from Google Earth Pro ^TM^ (Google Earth, 2019 Google image, 2019 CNES/Airbus, image 2019 Maxar Technology). B. Selected households from Google Earth Pro ^TM^ (Google Earths Image 2019 Terra metrics, 2019, Google).

GoogleEarth Pro ^TM^ Google Earth Pro version 7.3 (Google, Amphitheatre Pkwy, Mountain View, CA, USA). satellite imagery was used to digitize and enumerate all household structures in the village of Sussundenga ( Figure 2). This was a pilot study to determine malaria prevalence, risk factors, and health seeking behaviors. The sample size was determined by feasibility for the study team and study design of the community based cross-sectional survey. All households in the study area were digitized and enumerated using Google Earth Pro. With the aim of enrolling 100, a random sample of 125 households was taken, as backup for refusals and errors in the digitizing process (misclassified non-household structures). 

Coordinates of the households were extracted using a GPS device and maps of the selected households to conduct study visits. The study involved two visits to the selected households. The first was a notification visit where the study team introduced themselves to the head of the household and explained the objectives and procedures of the study. It is customary for the head of household to provide permission to the study team before any activities take place at the household involving other household members. Once the head of household gave permission, the study team conducted a household census with the head of household and begin the process of individual written informed consent with the household residents, for all adult (18+ years) residents and parental permission and consent from minors.

After obtaining consent from the household residents, the study team informed participants when they would return the following day to conduct the study activities. The only eligibility requirement was that the residents live in the household full time. Data collectors verbally administered a questionnaire to collect the basic demographics. The field study was carried out from December 2019 to January 2020.

The study nurse collected current malaria specific symptoms by self-report and took participant’s temperature using a digital thermometer (GP-300, RoHS:ISO 9000). They then collected a finger prick blood sample to administer a Rapid Diagnostic Test (RDT), RightSign Biotest ^R^ (Biotest, Hangzhou Biotest Biotech Co, China, Ref.No:IMPF – C51S). According to the manufacture, this test captures the HRP2 antigen on the strip and has a sensitivity is >99.0%. The results were recorded and, in the event, that a participant was positive for malaria, the study nurse referred them to the Sussundenga rural health center (RHC) for diagnosis confirmation and treatment. The questionnaire was conducted using tablet computers with the REDCap a secure, web-based data capture tool. Study data were collected and managed using REDCap electronic data capture tools hosted at University of Minnesota ^13^ downloaded to an Excel sheet for analysis. ^42^ REDCap (Research Electronic Data Capture) is a secure, web-based application designed to support data capture for research studies, providing: 1) an intuitive interface for validated data entry; 2) audit trails for tracking data manipulation and export procedures; 3) automated export procedures for seamless data downloads to common statistical packages; and 4) procedures for importing data from external sources.

### Data analysis

This study was a cross-sectional community-based survey. The analyses were conducted on datasets downloaded from REDCap to an Excel spread sheet. A binary variable was used to represent the dependent variable, malaria infection, to show whether malaria was present (positive) to RDT or absent (negative) was used. 

The explanatory variables analyzed were the following sociodemographic factors: age, if the person was an adult or child, age category, sex, history of malaria treatment, paid employment, cell phone ownership, education level, population density, location (neighborhood), household category and household size.

The malaria prevalence, was calculated by dividing positive cases of malaria by the study population tested at the time multiplied by 100. ^14^

$$\;\text{Prevalence}\;\left(\%\right)=\frac{\text{Persons having malaria}}{\text{Tested during the period}}\;\times \;100$$ (1)

Chi-square for proportion of age group and sex was tested. To establish the relationship between malaria prevalence and sociodemographic factors, logistic backward stepwise logistic regression was used with the following model:

$$X_{i}:g\;\left(\mathrm{Pi}\right)={ß}_{o}+{ß}_{1}x_{1}+{ß}_{2}x_{2+\ldots }\;{\mathrm{ßix}}_{i}$$ (2)

Where: *G (P _i_)* = link function

*P _i_* = likelihood of response for the -ith factor

*ß*_o_ = intercept

*ß _1_* = coefficient

*x _i_* = independent variable.

This method starts with a full (saturated) model and each step gradually eliminates variables that do not contribute. Allowing for a reduced model that best explains the data. This method is useful since, it reduces the number of predictors, reducing multicollinearity and resolves overfitting. ^15^

To evaluate potential confounders and, effect modifiers between the final model variables, the Hosmer–Lemeshow (1989) test was performed. ^16^ To build the final model, the independent variables *p*<0.05 were included. Outcomes such as scores statistic’s, regression coefficient’s, significance levels of variable coefficients and, overall classification accuracy were performed.

The sensitivity (conditional probability of a positive test given that the patient has malaria) of the final model measures the proportion of positive that were correctly identified and, was calculated using ^14^:

$$\text{Sensitivity}\left(\%\right)=\frac{\text{Number of true malaria positive}}{\left(\text{Number of true malaria positive}+\text{Number of false malaria negative}\right)}\;\times \;100$$ (3.1)

The specificity (conditional probability of a negative test given that the patient is well) of the final model measures the proportion of negative case correctly identified and was calculated using ^17^:

$$\text{Specificity}\left(\%\right)=\frac{\text{Number of true negatives}}{\left(\text{Number of true malaria negatives}+\text{Number of false malaria positives}\right)}\;\times \;100$$ (3.2)

Positive predictive value (PPV) that is, the conditional probability, whether the screened people who tested positive do or do not actually have malaria was calculated using ^17^:

$$\mathrm{PPV}\left(\%\right)=\frac{\text{Number of true malaria positive}}{\left(\text{Number of true malaria positive}+\text{Number of false malaria positive}\right)}\;\times \;100$$ (3.3)

Negative predicted value (NPV) that is, the conditional probability that an individual with a test indicative of no malaria infection is actually disease free, was calculated using ^17^:

$$\mathrm{NPV}\left(\%\right)=\frac{\text{Number of true malaria negatives}}{\left(\text{Number of true malaria negatives}+\text{Number of false malaria negative}\right)}\;\times \;100$$ (3.4)

All tests were carried out using SPSS IBM version 20 (IBM Corporation, Armonk, New York, USA) (RRID: SCR_002865).

## Results

### Malaria prevalence, sex, age and, age group and education level of participants

**Figure 3.**
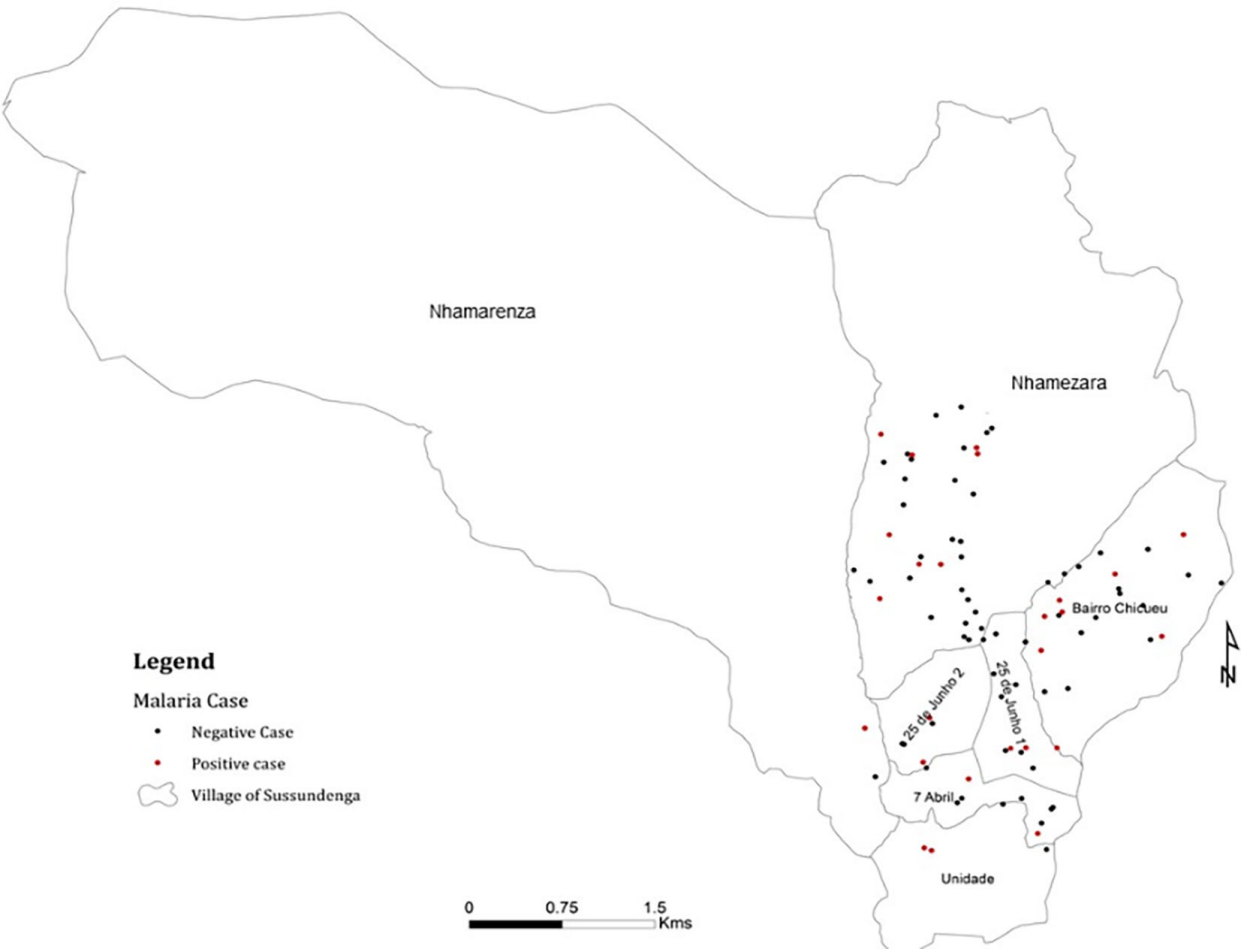
Malaria positive and negative cases in Sussundenga village.

From 125 selected households 100 were visited Figure 3 presents the positive and negative cases per visited site. Of the 358 participants tested and, interviewed 108 (31.6%) tested positive for malaria. There was an equal distribution of the enrolled participants among sex, 55% were female and 45% males, Chi-squared = 0.081, P = 0.8872, Degree of freedom (DF) = 1.

The age of participants varied from 1 to 80 years old, with a median of 17 years and an average of 21 standard deviation (SD), 16.2 years old. The participants’ education level varied, where 35.1% had no education or less than primary (5 grades), 47.4% had primary or basic school (grades 5 to 10) and 17.5% had secondary and higher education.

### Malaria prevalence by age category

**Figure 4.**
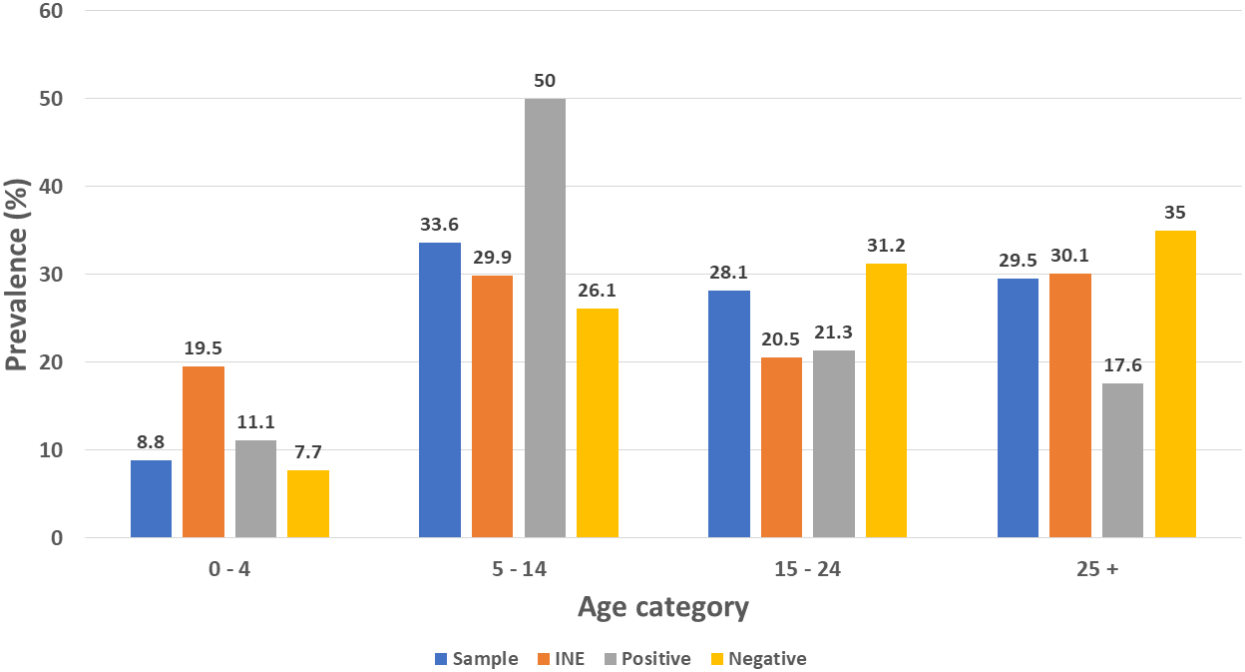
Malaria prevalence by age group in Sussundenga Village, INE = National Institute of Statistics.

Figure 4 presents the malaria positivity results for age categories. Half of the malaria positive cases occurred among those 5 to 14 years age category. This category comprises has 32.7% of the Sussundenga population according to the National Institute of Statistics (INE). The age category of over 24 years presented 17.6% of the malaria cases, this age category comprises 30.4% of the Sussundenga population according to the INE. There was a statistically significant difference in positive malaria cases among groups, chi-square = 17.527, P = 0.0075, Df = 6.

### Association between malaria infection and sociodemographic factors

The backward stepwise regression selection of predictors into the binary logistic model produced a series of models and, in this study, we only present the relevant, initial models and other outputs can be found in appendix 1.

Table 1 presents the backward stepwise (Wald) model summary and the Nagelkerke’s R ^2^ in final step is 0.135. This suggests that presence of malaria variation shown in the dependent variable of this model is approximately 13.5%. 

**Table 1.** Backward stepwise model summary.

Step	-2 Log likelihood	Cox & Snell R Square	Nagelkerke R Square
1	408.482 ^a^	.109	.151
9	413.304 ^b^	.096	.135

^a^ Estimation terminated at iteration number 5 because parameter estimates changed by less than .001.

^b^ Estimation terminated at iteration number 4 because parameter estimates changed by less than .001.

Table 2 presents the Hosmer and Lemeshow test, indicating that this model fit the data.

**Table 2.** Hosmer–Lemeshow test.

Step	Chi-squared	DF	Sig.
1	8.558	8	.381
9	5.990	8	.648

Df = degrees of freedom, Sig. = Wald’s.

Table 3 presents the classification table of the final model, that is, the models capability to predict malaria positive cases, indicating a model accuracy of 71.6%. The sensitivity of the final model in classifying malaria positive cases is 73.3% and specificity of the final model to classify malaria negative cases is 93.3%. The positive predictive value is 66% and, the negative predictive value is 72.5% meaning that, the final model is able to predict 66% of malaria positive tests and, 72.5% negative malaria tests.

**Table 3.** Final backward stepwise (Wald) model classification table.

Observed			Predicted		
			Malaria results		Percentage correct
			Negative	Positive	
Step 1	Malaria result	Negative	218	22	90.8
		Positive	77	39	33.6
	Overall percentage				72.2
Step 9	Malaria result	Negative	224	16	93.3
		Positive	85	31	26.7
	Overall percentage				71.6

Wald = Wald test.

^a^ The cut-off value is .500.

Table 4 presents the Wald’s test of significance and the odds ratio predictors variables in the final model. From the results, pervious malaria treatment ( *p*=0.15), population density ( *p*=0.05), and age group ( *p*=0.00) were significant predictors while, household category did not add significantly to the model. The table indicates that the age category 0 to 4 years old as almost three times more likely to test positive for malaria (OR 2.829, 95% CI 1.153–6.944), 3.6 times (OR 3.61, 95% CI 1.952 – 6.755) for age group 5 to 14 years and, 1.6 times for the age group of 15 or older (OR 1.603, 95% CI 0.824 – 3.117).

**Table 4.** Final model Wald’s of significance and odds ratio of predictor variables.

		Constant (B)	S.E.	Wald	DF	Sig.	Exp(B)	95% CI for EXP(B)	
								Lower	Upper
Step 9 ^a^	Previous malaria treatment	−.607	.249	5.941	1	.015	.545	.335	.888
	Population density	.000	.000	3.830	1	.050	1.000	1.000	1.000
	Household category	−.601	.315	3.651	1	.056	.548	.296	1.016
	Age category			18.890	3	.000			
	Age category (0 to 4 years)	1.040	.458	5.155	1	.023	2.829	1.153	6.944
	Age category (5 to 14 years)	1.289	.317	16.573	1	.000	3.631	1.952	6.755
	Age category (> 14 years)	.472	.339	1.934	1	.164	1.603	.824	3.117
	Constant	−.821	.305	7.232	1	.007	.440		

*B =* regression coefficients, S. E = standard errors, Wald = Wald test, Df = degrees of freedom, Sig. = Wald’s significance, *Exp(B) (OR =* odds ratio, 95% CI = confidence interval of the odds ratio.

^a^ Variable(s) entered on step 1: Adult or child, Sex, Previous malaria treatment, Employment, Age, Cell phone, Education, Population density, Household size, HH category, Age category, Location.

The built model is:

$$g\;\left(\mathrm{Pi}\right)=-.821-.607\;\text{Previous malaria treatment}+1.040\;\mathrm{Age}\;\text{category}\;\left(0\;\text{to}\;4\;\text{years}\right)+1.289+\mathrm{Age}\;\text{category}\;\left(5\;\text{to}\;14\;\text{years}\right).$$

## Discussion

In this study, malaria prevalence was 31.6% for Sussundenga Village, much higher than the prevalence recorded in Chimoio city (20.1%). ^18^ In the neighboring Zimbabwe, malaria prevalence was 19.5% in Mutare and 50.9% in Mutasa districts in 2016. ^19^ In southern Zambia a study in 2020, reported parasite prevalence between 0.7 and 1.8% ^20^ and, 34% in Malawi in 2016. ^21^

No significant difference was found between different sexes in this study. Similar results were reported in Chimoio, Mozambique in 2018, ^18^ in Malawi in 2020 ^22^ and in Zimbabwe ^23^ in 2021.

This study recorded half of the malaria prevalence in the 5 to 14 years age category and, an odds ratio of 3.61. In Ghana this age groups accounted for 43.3% and, in Rwanda the odds of infection by malaria were reported to be 1.817 times for this age category. ^24^^,^^25^ Studies in Kenya indicated that highest malaria prevalence occurs in children between ages of 11 to 14 and, children from 5 to 18 years as the most at-risk age category. ^26^^,^^27^ Contrarily, in Chimoio, Mozambique it was reported 52% of malaria cases are found in children under five, ^18^ this discrepancy may due to the fact that the present study was carried out at community level while, the Chimoio study was carried out from health center data.

This study suggests that recent diagnosis and treatment for malaria infection reduces the odds of subsequent infection approximately by 54.5%. Similar results were reported in Mozambique, Ghana, Comoros, Kenya, Indonesia and India. ^28^^–^^34^ This reduction in odds is likely due to prophylactic effect of ACT. It provides protection from 2 weeks to 1 month after completion. After repeated infections, the individual develops a certain degree of immunity. Also, when re-infected, patients tend to present a mild form of the diseases without symptoms and, natural active immunity is established after ten or more *P. falciparum* infections, which can be sufficient to suppress symptoms and clinical signs. ^35^

Different results were reported in Angola where women who had a previous malaria infection during pregnancy also had a higher risk to contract malaria. ^36^ This is likely because pregnant women may take sulfadoxine-pyrimethamine rather than ACT.

In this study population density was found as a significant predictor for an individual to test positive for malaria. Similar results were reported in Chimoio ^18^ in 2016, in a study in 14 endemic African countries ^37^ in 2017 and in Ethiopia ^38^ in 2015.

The variables age, if the person was an adult or child, sex, paid employment, cell phone ownership, education level, location (Bairro) and household size were removed from the model due to redundancy and for not adding significance to the model.

The age category is a good proxy for age group and, household size for household category. Paid employment and cell phone ownership variables were included in this study, as rural wealth indicators. These were not found significant predictors contrary to a study in Mozambique that indicated that, children from higher income families (58%) tend to be at lower risk for malaria compared to children from lower income families (43%). ^39^ Another study in sub-Saharan Africa ^40^ showed that, malaria prevalence increases with a decrease in income in 2018.

Education level was not finding significant predictor in this study. Similar results were reported in Malawi in 2018, ^41^ Indonesia and India. ^33^^,^^34^ There were conflicting results reported in Mozambique ^39^ in 2011, in Ghana in 2014 and in Sub-Saharan Africa ^40^ in 2018.

In this study it is suggested that approximately 13.5% of the variation in malaria infection can be attributed to sociodemographic and economic traits. A previous study modelled the influence of climate on malaria occurrence in Chimoio and indicated that environmental traits accounted for 72.5% of malaria occurrences. ^7^ This implies that non-environmental factors such as sociodemographic, economic, cultural and behavioral traits could partially account for the remaining percentage, consistent with the present study.

The capability model using social, economic, and demographic variables to predict malaria positive cases (model accuracy), was 72.3% in this study. A logistic regression model analyzing hematological parameter and age in Ghana reported 77.4%. ^24^ The sensitivity of the final model in classifying malaria positive cases was 73.3% and the final model was able to predict 66% (PPV) meaning that the model is very effective in predicting malaria infection using sociodemographic characteristics. In Iran a model predicting malaria re-introduction reported 81.8% positive predictive value ^41^ and 52.72% in Ghana in a model analyzing hematological parameter and age. ^27^

### Limitations of the study

Data collection for this study was conducted in December and January during the rainy and wet season which is also the peak malaria transmission season. Because of this, it is likely that we detected a large number of infections and results reflect this season and my not be representative of malaria dynamics in the dry season. The RightSign Biotest ^R^ test detects the histidine rich protein 2 antigen of the *P. falciparum* parasite which can last over a month in the blood among patients recently treated with malaria.

## Conclusion

This study evaluated the sociodemographic factors that affect malaria prevalence in Sussundenga Village, Mozambique. Recent diagnosis and treatment, population density and age category were found to be significant predictors. The model accuracy was 72.3% implying that the model is robust. Targeting malaria control at the community level can contribute to decreased transmission that may be more impactful than passive case detection and treatment alone. This model indicates that 13.5% of malaria cases can be attributed to sociodemographic factors while previous studied indicated that environmental conditions are attributed to approximately 73% of malaria cases. Further studies are needed especially in the dry season and in other areas of the district to fully understand the malaria transmission dynamics in this region and inform efficient control measures.

## Data availability

### Underlying data

Harvard Dataverse: Replication Data for: Modelling sociodemographic factors that affect malaria prevalence in Sussundenga, Mozambique: a cross-sectional study. https://doi.org/10.7910/DVN/BUMDEM. ^42^

This project contains the following underlying data:

- [Aditional file -F1000Research.tab] (raw data from questionnaires).

Data are available under the terms of the Creative Commons Zero “No rights reserved” data waiver (CC0 1.0 Public domain dedication).

## Ethical consideration

This study is part of the Malaria Risk, Prevention, and Health Seeking Behaviors in Sussundenga, Mozambique Project. All participants, or the guardians provided informed written assent and consent prior to participation. Ethical review and approval for the study was completed by the Institutional Review Board (IRB) at the University of Minnesota [STUDY00007184] and from A Comissão Nacional de Bioética em Saúde (CNBS) at the Ministry of Health of Mozambique [IRB00002657]. The images taken from google and other sites were properly cited and referenced.
