# Supplementary material for: Modelling sociodemographic factors that affect malaria prevalence in Sussundenga, Mozambique: a cross-sectional study
Source: F1000Res. 2022 May 5;11:185. Originally published 2022 Feb 14. [Version 2] doi: 10.12688/f1000research.75199.2 (PMC9131438; doi:10.12688/f1000research.75199.2)
Supplement: Peer review history

# Peer review: Reviewer response for version 2

**DOI:** 10.5256/f1000research.133640.r136728

**Published:** 2022-05-19

**Authors:** Gabriel Zorello Laporta

**Affiliations:** 1 Graduate Research and Innovation Program, Centro Universitario FMABC, Santo André, Brazil

I want to thank the authors by the responses that clarified my doubts about this intellectual piece.

Is the work clearly and accurately presented and does it cite the current literature?

Yes

If applicable, is the statistical analysis and its interpretation appropriate?

Partly

Are all the source data underlying the results available to ensure full reproducibility?

Partly

Is the study design appropriate and is the work technically sound?

Partly

Are the conclusions drawn adequately supported by the results?

No

Are sufficient details of methods and analysis provided to allow replication by others?

Partly

Reviewer Expertise:

Epidemiology of vector-borne diseases

I confirm that I have read this submission and believe that I have an appropriate level of expertise to confirm that it is of an acceptable scientific standard.

# Peer review: Reviewer response for version 2

**DOI:** 10.5256/f1000research.133640.r136727

**Published:** 2022-05-17

**Authors:** Ewan Cameron

**Affiliations:** 1 School of Public Health, Faculty of Health Sciences, Curtin University, Perth, WA, Australia

I thanks the authors for their detailed and considerate responses to my first round reviewer questions/comments. I am happy to recommend the revised article for indexing.

Is the work clearly and accurately presented and does it cite the current literature?

Partly

If applicable, is the statistical analysis and its interpretation appropriate?

Yes

Are all the source data underlying the results available to ensure full reproducibility?

Yes

Is the study design appropriate and is the work technically sound?

Partly

Are the conclusions drawn adequately supported by the results?

Partly

Are sufficient details of methods and analysis provided to allow replication by others?

Yes

Reviewer Expertise:

Malaria risk stratification; statistics; mechanistic disease modelling; model calibration

I confirm that I have read this submission and believe that I have an appropriate level of expertise to confirm that it is of an acceptable scientific standard.

# Peer review: Reviewer response for version 1

**DOI:** 10.5256/f1000research.79031.r129856

**Published:** 2022-04-14

**Authors:** Gabriel Zorello Laporta

**Affiliations:** 1 Graduate Research and Innovation Program, Centro Universitario FMABC, Santo André, Brazil

Ferrao *et al*. provide us with a cross-sectional study aimed at estimation of malaria falciparum prevalence in Mozambique, Africa. The study is well written and has good data, but some parts are not clear enough, as follows:

1. Study sample size and figure 2. Please specify the randomization process to select 125 households in the study area. What is the statistical power of the selection of 100 houses? If the study area’s landscape is heterogenous in terms of risk of malaria infection, is a random selection the best approach for selection? Should a stratified sample selection approach be used instead?
2. To estimate sensitivity and specificity, it is essential to have a training dataset and a testing dataset. The training dataset is to build a statistical model and the testing dataset is to evaluate the fitted model. Please specify the training and the testing data.
3. The built model shows that access to treatment and age are the only important predictors. Please explain the lack of importance of social and economic predictors in the built model. The built model shows a coefficient (1.289) lacking a variable. Please revise.
4. RDT based on HRP2 has issues to detect falciparum lacking HRP2 genes. This is an important limitation and indicates that the estimates of prevalence may be underestimated.
5. In data source, please include labels to the variables’ levels per variable, i.e., 1=, 0=.
6. Conclusions have several issues:
  “Recent diagnosis and treatment, population density and age category were found to be significant predictors”. Issue: pop density was not significant predictor.
  “The model accuracy was 72.3% implying that the model is robust”. Issue: it depends on the approach that it was calculated.
  “This model indicates that 13.5% of malaria cases can be attributed to sociodemographic factors while previous studied indicated that environmental conditions are attributed to approximately 73% of malaria cases”. Issue: be specific of which sociodemographic factor, and environmental conditions were not studied in this study.

Is the work clearly and accurately presented and does it cite the current literature?

Yes

If applicable, is the statistical analysis and its interpretation appropriate?

Partly

Are all the source data underlying the results available to ensure full reproducibility?

Partly

Is the study design appropriate and is the work technically sound?

Partly

Are the conclusions drawn adequately supported by the results?

No

Are sufficient details of methods and analysis provided to allow replication by others?

Partly

Reviewer Expertise:

Epidemiology of vector-borne diseases

I confirm that I have read this submission and believe that I have an appropriate level of expertise to confirm that it is of an acceptable scientific standard, however I have significant reservations, as outlined above.

# Response

**Published:** 2022-04-29

**Authors:** Joao Ferrao

**Affiliations:** Instituo Superior de Ciencias de Educacao a Distancia, Mozambique

Dear Reviewer. thank you very much for your precious comments. They are very useful and they were used to improve the manuscript.

- Ferrao *et al*. provide us with a cross-sectional study aimed at estimation of malaria falciparum prevalence in Mozambique, Africa. The study is well written and has good data, but some parts are not clear enough, as follows:
  Study sample size and figure 2.
  Please specify the randomization process to select 125 households in the study area., what is the statistical power of the selection of 100 houses? If the study area’s landscape is heterogenous in terms of risk of malaria infection, is a random selection the best approach for selection? Should a stratified sample selection approach be used instead?

**Response:** Thanks for the important question raised. Indeed, this was a pilot study to determine malaria prevalence, risk factors, and health seeking behaviors. The sample size was determined by feasibility for the study team and study design of the community based cross-sectional survey.

 All households in the study area were digitized and enumerated using Google Earth Pro. A random sample of 125 households was taken, with the aim of enrolling 100 to account for errors in the digitizing process.

 The village is relatively small (156.9 Km ^2^) and we added the area of the village in the test. The Sussundenga village is within an area of 156.9 Km ^2^.  

  

- To estimate sensitivity and specificity, it is essential to have a training dataset and a testing dataset. The training dataset is to build a statistical model and the testing dataset is to evaluate the fitted model. Please specify the training and the testing data.

**Response: **Thank you very much for the raised question. Usually the prediction of classes for data classification are based on finding the optimum boundary between classes.

 For this case where we used an accuracy with cut-off=0.5, we don’t see the need of training data.

 After data imputation and engendering feature the third step was to split data into train and test. This was carried out by the software.

  

- The built model shows that access to treatment and age are the only important predictors. Please explain the lack of importance of social and economic predictors in the built model.

**Response: **Thank you very much for the question. Several studies indicated that the major causes of malaria occurrence are the climatic conditions specially temperature bellow 20 ^o^C the sporogony cease, and humidity below 50 and over 90 %.

  

- The built model shows a coefficient (1.289) lacking a variable. Please revise.

**Response:** Thank you very much for the observation. This was revised, the variable is Age category (5 to 14 years).

  

- RDT based on HRP2 has issues to detect falciparum lacking HRP2 genes. This is an important limitation and indicates that the estimates of prevalence may be underestimated.

**Response:** This is true and a limitation of the current HRP2 based RDTs. There are limited data on HRP2 deletions throughout Mozambique and specifically in Manica Province. However, in a study published in 2019 ( https://www.ncbi.nlm.nih.gov/pmc/articles/PMC6711899/) the authors found very few cases of HRP2 deletion and impacts on the efficacy of the current RDTs. Of those infections not detected by the RDTs, not all were *P. falciparum*. It is unlikely in our study setting that HRP2 deletions impacted the efficacy of the RDT and biased our prevalence estimates.

  

- In data source, please include labels to the variables’ levels per variable, i.e., 1=, 0=,II)
- **Conclusions have several issues:**
  “Recent diagnosis and treatment, population density and age category were found to be significant predictors”. Issue: pop density was not significant predictor.

**Response:** Thank you for the observation. Rescaled to 3 decimal places its significant. This was corrected in table.  

  

- “The model accuracy was 72.3% implying that the model is robust”. Issue: it depends on the approach that it was calculated.

**Response:** Thanks for the observation.

  

- “This model indicates that 13.5% of malaria cases can be attributed to sociodemographic factors while previous studied indicated that environmental conditions are attributed to approximately 73% of malaria cases”. Issue: be specific of which sociodemographic factor, and environmental conditions were not studied in this study.

**Response:** Thank you very much for the observation. In the manuscript, more text was added to address this issue.

# Peer review: Reviewer response for version 1

**DOI:** 10.5256/f1000research.79031.r127464

**Published:** 2022-04-01

**Authors:** Ewan Cameron

**Affiliations:** 1 School of Public Health, Faculty of Health Sciences, Curtin University, Perth, WA, Australia

In the manuscript entitled, "Modelling sociodemographic factors that affect malaria prevalence in Sussundenga, Mozambique: a cross-sectional study", the authors present the results of a survey and statistical analysis designed to uncover predictors of positive parasite status by rapid diagnostic test in this area of endemic malaria transmission. Malaria is a significant contributor to disease burden in Mozambique, so this topic is of high importance. In my report below I raise a number of questions for the authors regarding aspects of the analysis that were unclear to me (i.e., perhaps where the clarity of presentation could be improved) or where I felt that further analysis might strengthen the conclusions.

 Context and Aims:

- The framing of the study in terms of context and aims as it currently stands deserves considerable review. Owing precisely to its status as one of the most malarious countries in the world, Mozambique has attracted a great deal of attention from malaria researchers over many decades and there have been many studies investigating environmental and socio-demographic factors behind malaria transmission in the country. For example, Temu *et al*. (Plos One, 2012) ^1^ identified IRS use, pig-keeping, and house construction as key factors influencing malaria risk in Zambezia province. See also, Brentlinger *et al*. (Am J Trop Med Hyg, 2007) ^2^. Likewise, there are many studies examining factors related to explanatory factors for malaria prevalence such as treatment seeking (Cassy *et al.* Mal J, 2019) ^3^ and ITN use (Moon *et al.*, Mal J, 2016) ^4^. Factors shaping malaria risk in Mozambique have also recently been studied in the context of measuring the impact of malaria control strategies in Mozambique by Galatas *et al.* (Plos Medicine, 2020) ^5^.  
  This wealth of prior research and understanding contrasts strikingly with the authors' proposition that "little is known about the epidemiology to inform appropriate and effective interventions". Importantly, it also raises the question of why information on two well-known factors shaping malaria transmission in Mozambique, namely IRS and ITN use, do not seem to have been captured or included in this study? Another key known factor, household construction, may have been included but there is no detail that I could see in the manuscript to illuminate the meaning of the "household category" variable used in the model?
- Regarding the statement "ITNs are only available at antenatal clinics, indicated for pregnant women and children under five": I'm not sure that this is well phrased, since the WHO recommends universal net use in high transmission areas. While ITN distribution campaigns focus on the highest risk groups of young children and pregnant women these are not the only groups who should be advised to use bed nets; likewise, ITNs would generally be available commercially at markets.  (See e.g. Scott *et al*. Mal J, 2021) ^6^.

Statistical Analysis:

- The statistical analysis method used to derive the primary results of this study, namely the identification of key factors behind malaria prevalence in the study area, is a stepwise logistic regression, which is indeed appropriate for this objective. Some minor details require clarification or revision: 
  (i) there are some minor formatting errors in the equations: e.g. on page 5, equation 2 uses lower case g and then an upper case G for seemingly the same function, and betai should have a subscript i as in beta_i; on page 8 the last equation should have 1.289 "x" Age category (5 to 14 years) instead of "+"
  (ii) "To evaluate potential confounders and, effect modifiers between the final model variables, the Hosmer–Lemeshow (1989) test was performed." This doesn't make sense to me: the HM test is for model specification / acceptable fit, rather than for breaking variables down into their roles in the causal hierarchy.
  (iii) I was confused by the chi square test reports in some places: for the distribution by sex the chi-squared statistic of 0.081 doesn't sound like the right order of magnitude and in fact I get 0.081 as the p-value for a binomial exact test on this sample so perhaps this is a typo?; for the tests by age category, since this is a four x two table I would have thought we're looking at 3 degrees of freedom rather than 6?
  (iv) The population density variable must have a large dynamic range because it is assigned a slope ("constant"?) of 0.000 in table 8: could this be rescaled so that we can see its slope within the 3 decimal places?
  (v) I was confused why the age categories changed from four in the earlier discussion to three in Table 4? Also, it would help to nominate one age group as the reference group so that the odds ratios can be understood as relative to that group.
  (vi) I'm confused by the focus on understanding the predictive accuracy of the model in terms of specificity and sensitivity(*), which are appropriate for a diagnostic tool, but which may not be particularly relevant to the use of a risk factor model such as this one for field epidemiology. I.e., if the end use is to prioritise the delivery of a particular intervention such as seasonal malaria chemopraxis then identifying that a certain age group has twice the parasite prevalence of another could be of substantial value even where the sensitivity was low because prevalence itself was very low across both strata. This comes back to the context and aims of the study, in the sense that the value of the fitted model (or more precisely the knowledge discovered through it) is ultimately something that exists only relative to the way in which it is intended to be used.
  (*also: are these defined according to a thresholding of the predictive prevalence above and below 50%?)

Is the work clearly and accurately presented and does it cite the current literature?

Partly

If applicable, is the statistical analysis and its interpretation appropriate?

Yes

Are all the source data underlying the results available to ensure full reproducibility?

Yes

Is the study design appropriate and is the work technically sound?

Partly

Are the conclusions drawn adequately supported by the results?

Partly

Are sufficient details of methods and analysis provided to allow replication by others?

Yes

Reviewer Expertise:

Malaria risk stratification; statistics; mechanistic disease modelling; model calibration

I confirm that I have read this submission and believe that I have an appropriate level of expertise to confirm that it is of an acceptable scientific standard, however I have significant reservations, as outlined above.

## References

[1] : High prevalence of malaria in Zambezia, Mozambique: the protective effect of IRS versus increased risks due to pig-keeping and house construction. *PLoS One* .2012;7(2) : 10.1371/journal.pone.0031409 e31409 10.1371/journal.pone.0031409 22363640PMC3282725

[2] : Intermittent preventive treatment of malaria during pregnancy in central Mozambique. *Bull World Health Organ* .2007;85(11) : 10.2471/blt.06.033381 873-9 10.2471/blt.06.033381 18038078PMC2636267

[3] : Care-seeking behaviour and treatment practices for malaria in children under 5 years in Mozambique: a secondary analysis of 2011 DHS and 2015 IMASIDA datasets. *Malar J* .2019;18(1) : 10.1186/s12936-019-2751-9 115 10.1186/s12936-019-2751-9 30940127PMC6444821

[4] : Factors associated with the use of mosquito bed nets: results from two cross-sectional household surveys in Zambézia Province, Mozambique. *Malar J* .2016;15: 10.1186/s12936-016-1250-5 196 10.1186/s12936-016-1250-5 27068575PMC4827200

[5] : A multiphase program for malaria elimination in southern Mozambique (the Magude project): A before-after study. *PLoS Med* .17(8) : 10.1371/journal.pmed.1003227 e1003227 10.1371/journal.pmed.1003227 32797101PMC7428052

[6] : Factors associated with use of insecticide-treated net for malaria prevention in Manica District, Mozambique: a community-based cross-sectional survey. *Malar J* .2021;20(1) : 10.1186/s12936-021-03738-7 200 10.1186/s12936-021-03738-7 33906642PMC8077836

# Response

**Published:** 2022-04-29

**Authors:** Joao Ferrao

**Affiliations:** Instituo Superior de Ciencias de Educacao a Distancia, Mozambique

Dear Reviewer. thank you very much for your precious comments. They are very useful and they were used to improve the manuscript.

- In the manuscript entitled, "Modelling sociodemographic factors that affect malaria prevalence in Sussundenga, Mozambique: a cross-sectional study", the authors present the results of a survey and statistical analysis designed to uncover predictors of positive parasite status by rapid diagnostic test in this area of endemic malaria transmission. Malaria is a significant contributor to disease burden in Mozambique, so this topic is of high importance.

**Response:** Thank you very much for the comment.

  

- In my report below, I raise a number of questions for the authors regarding aspects of the analysis that were unclear to me (i.e., perhaps where the clarity of presentation could be improved) or where I felt that further analysis might strengthen the conclusions.

**Response:** Thank you very much. All comments will be taken in consideration accordingly.

**Context and Aims:**

- The framing of the study in terms of context and aims as it currently stands deserves considerable review.

**Response:** Thank you very much for the comment. The review will be done according to the reviewer's suggestions.

  

- Owing precisely to its status as one of the most malarious countries in the world, Mozambique has attracted a great deal of attention from malaria researchers over many decades and there have been many studies investigating environmental and socio-demographic factors behind malaria transmission in the country. For example, Temu *et al*. (Plos One, 2012) ^1^ identified IRS use, pig-keeping, and house construction as key factors influencing malaria risk in Zambezia province. See also.

**Response:** Thank you very much for the comment. These studies were considered in our discussion. Considering the size of the country 800,000 Km2, more than 2,700 Km long, its heterogeneity in terms of landscape, sociodemographic and culture and poor resources, I would consider that the number of studies “investigating environmental and socio-demographic factors behind malaria transmission in the country” very few for local programmatic decisions making policies.

  

- See also Brentlinger *et al*. (Am J Trop Med Hyg, 2007).

**Response:** Thanks for the recommendation. We find two studies from the recommended authors. However, we feel that they are specific for HIV patients and are outdated. 

  

- Likewise, there are many studies examining factors related to explanatory factors for malaria prevalence such as treatment seeking (Cassy *et al.* Mal J, 2019) and ITN use (Moon *et al.*, Mal J, 2016).

**Response:** Thanks very much for the observations. Two articles (2019 and 2022 on care seeking were found and include in our discussion. As stated in the paper “This study is part of the Malaria Risk, Prevention, and Health Seeking Behaviors in Sussundenga, Mozambique Project”. There is another study dealing specifically with this issue.  

  

- Factors shaping malaria risk in Mozambique have also recently been studied in the context of measuring the impact of malaria control strategies in Mozambique by Galatas *et al.* (Plos Medicine, 2020) ^5^ .  

**Response:** Thank you very much for the comment. The studies were considered in our discussion.

  

- This wealth of prior research and understanding contrasts strikingly with the authors' proposition that "little is known about the epidemiology to inform appropriate and effective interventions". Importantly, it also raises the question of why information on two well-known factors shaping malaria transmission in Mozambique, namely IRS and ITN use, do not seem to have been captured or included in this study?

**Response:** Thank you very much for rising a very important issue of IRS and ITN use. We do consider IRS and ITN use suiting better as “health seeking behaviors” and due to its high relevance, a separate paper was produced: https://www.researchgate.net/publication/357008627_P_Falciparum_Community_Prevalence_and_Health_Seeking_Behaviors_in_Rural_Sussundenga_District_Mozambique

  

- Another key known factor, household construction, may have been included but there is no detail that I could see in the manuscript to illuminate the meaning of the "household category" variable used in the model?

**Response:** Thank you very much for the question.

 Household size and household construction are very important variables. In the methodology we rephrase the variables and their meaning. We hope that now is clear that household category means type of house or type of construction. In this study, household category or household construction was found as a predictor variable.

  

- Regarding the statement "ITNs are only available at antenatal clinics, indicated for pregnant women and children under five": I'm not sure that this is well phrased, since the WHO recommends universal net use in high transmission areas. While ITN distribution campaigns focus on the highest risk groups of young children and pregnant women these are not the only groups who should be advised to use bed nets; likewise, ITNs would generally be available commercially at markets.  (See e.g. Scott *et al*. Mal J, 2021) ^6^ .

**Response: **Thank you for the comment. We do agree that “young children and pregnant women are not the only groups who should be advised to use bed nets; Indeed, recent studies are indicating an age shift in Malaria due this situation.

 As for the statement: “likewise, ITNs would generally be available commercially at markets. We would agree in a “normal” market driven country. For the Mozambican case where, most people are living bellow the poverty line, buying a mosquito net for prevention can be a luxury.

 For example, in 2021, a mosquito factory in Chimoio, Manica closed it is doors and the major reason was lack of clients to purchase the nets.

 We added this useful contribution in our discussion.

**Statistical Analysis:**

- The statistical analysis method used to derive the primary results of this study, namely the identification of key factors behind malaria prevalence in the study area, is a stepwise logistic regression, which is indeed appropriate for this objective. Some minor details require clarification or revision: 

**Response:** Thank you very much for the observation.

  

- there are some minor formatting errors in the equations: e.g. on page 5, equation 2 uses lower case g and then an upper-case G for seemingly the same function,

**Response:** Thank you very much for observation. This was corrected.

  

- and betai should have a subscript i as in beta_i;

**Response:** Thank you for the observation. Correction was made.

  

- on page 8 the last equation should have 1.289 "x" Age category (5 to 14 years) instead of "+"

**Response:** Thank you for the observation.

  

- "To evaluate potential confounders and, effect modifiers between the final model variables, the Hosmer–Lemeshow (1989) test was performed." This doesn't make sense to me: the HM test is for model specification / acceptable fit, rather than for breaking variables down into their roles in the causal hierarchy.

**Response: **Thanks for a very good observation We agree, to avoid confusing we rephrased the sentence.

  

-  (iii) I was confused by the chi square test reports in some places: for the distribution by sex the chi-squared statistic of 0.081 doesn't sound like the right order of magnitude and in fact I get 0.081 as the p-value for a binomial exact test on this sample so perhaps this is a typo?;

**Response**: Thank you for the observation.

 As stated in the methodology, “Sussundenga has an estimated population of 31,429 inhabitants, 47% males and 53% females”. In the present study, the enrolled participants among sex, 55% were female and 45% males. Using the Biostat 5.3 software we find the following out put



 The table was corrected.

  

- For the tests by age category, since this is a four x two table I would have thought we're looking at degrees of freedom rather than 6?

**Response:** Thanks for the observation.

 We believe that is more appropriate to check the age category compared also to sample results and National Institute of Statistics projections for accuracy, giving us a 4 x 4 contingency table. The following recalculations are presented and were corrected in the manuscript.



 The table was corrected.

- The population density variable must have a large dynamic range because it is assigned a slope ("constant"?) of 0.000 in table 8: could this be rescaled so that we can see its slope within the 3 decimal places?

**Response:** This was very important observation. We increased one decimal place and we rewrite the equation.

  

- I was confused why the age categories changed from four in the earlier discussion to three in Table 4?

**Response:** Thank you for your valuable observation.

 We stated four age categories, 0 to 4, 5 to 14, 14 to 24 and > 24 (Additional file 1). The software did not find difference between age categories 14 to 24 and > 24 and grouped them as the same category. This seems to be right.

  

- Also, it would help to nominate one age group as the reference group so that the odds ratios can be understood as relative to that group.

**Response:** Thank you for the observation. We fill that a priori, it would be difficult to select a reference age group.

  

- I'm confused by the focus on understanding the predictive accuracy of the model in terms of specificity and sensitivity(*), which are appropriate for a diagnostic tool, but which may not be particularly relevant to the use of a risk factor model such as this one for field epidemiology. I.e., if the end use is to prioritise the delivery of a particular intervention such as seasonal malaria chemopraxis then identifying that a certain age group has twice the parasite prevalence of another could be of substantial value even where the sensitivity was low because prevalence itself was very low across both strata. This comes back to the context and aims of the study, in the sense that the value of the fitted model (or more precisely the knowledge discovered through it) is ultimately something that exists only relative to the way in which it is intended to be used.

**Response:** Thanks for the observation.

 Since sensitivity and specificity are measures of performance of a binary model, is pertinent to access them in the logistic model. We included an explanation in the methodology and we also discussed the results.

  

- (*also: are these defined according to a thresholding of the predictive prevalence above and below 50%?)

**Response: **Yes
